# Research on the Segmentation of Biomarker for Chronic Central Serous Chorioretinopathy Based on Multimodal Fundus Image

**DOI:** 10.1155/2021/1040675

**Published:** 2021-09-03

**Authors:** Jianguo Xu, Jianxin Shen, Qin Jiang, Cheng Wan, Zhipeng Yan, Weihua Yang

**Affiliations:** ^1^College of Mechanical and Electrical Engineering, Nanjing University of Aeronautics and Astronautics, Nanjing 210016, China; ^2^The Affiliated Eye Hospital of Nanjing Medical University, Nanjing 210029, China; ^3^College of Electronic and Information Engineering, Nanjing University of Aeronautics and Astronautics, Nanjing 210016, China

## Abstract

At present, laser surgery is one of the effective ways to treat the chronic central serous chorioretinopathy (CSCR), in which the location of the leakage area is of great importance. In order to alleviate the pressure on ophthalmologists to manually label the biomarkers as well as elevate the biomarker segmentation quality, a semiautomatic biomarker segmentation method is proposed in this paper, aiming to facilitate the accurate and rapid acquisition of biomarker location information. Firstly, the multimodal fundus images are introduced into the biomarker segmentation task, which can effectively weaken the interference of highlighted vessels in the angiography images to the location of biomarkers. Secondly, a semiautomatic localization technique is adopted to reduce the search range of biomarkers, thus enabling the improvement of segmentation efficiency. On the basis of the above, the low-rank and sparse decomposition (LRSD) theory is introduced to construct the baseline segmentation scheme for segmentation of the CSCR biomarkers. Moreover, a joint segmentation framework consisting of the above method and region growing (RG) method is further designed to improve the performance of the baseline scheme. On the one hand, the LRSD is applied to offer the initial location information of biomarkers for the RG method, so as to ensure that the RG method can capture effective biomarkers. On the other hand, the biomarkers obtained by RG are fused with those gained by LRSD to make up for the defect of undersegmentation of the baseline scheme. Finally, the quantitative and qualitative ablation experiments have been carried out to demonstrate that the joint segmentation framework performs well than the baseline scheme in most cases, especially in the sensitivity and F1-score indicators, which not only confirms the effectiveness of the framework in the CSCR biomarker segmentation scene but also implies its potential application value in CSCR laser surgery.

## 1. Introduction

**Figure 1:**
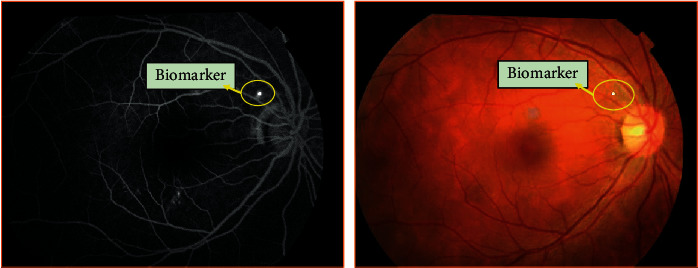
The biomarkers on the angiography and color fundus images.

CSCR is a macular disease which is characterized by neurosensory retinal detachment (NRD) with or without pigment epithelium detachment (PED) [1–3] and may result in blurred vision, central scotoma, deformed vision, and even permanent visual loss in serious cases. In general, the CSCR can be divided into acute and chronic types [4, 5], among which most acute CSCR patients can be self-healing within 3-4 months without any treatment [6]. However, the chronic cases are difficult to automatically return to normal and have to rely on surgery or drug intervention to block the deterioration of the course. In recent years, the laser surgery intervention has become one of the important means of CSCR therapy, which plays an effective role in inhibiting the development of CSCR. The traditional laser photocoagulation and micropulse laser photocoagulation are commonly used in CSCR laser surgery. The former employs a laser spot with a diameter of 50 to 100 *μ*m to directly act on the leakage area (i.e., the biomarkers), which can block the leakage outlet smoothly. Compared with the former, the latter has a wider range of work, which covers the entire CSCR edema area based on the multipoint scanning mode. In this paper, we mainly focus on the former laser surgery scheme, where the fast and accurate segmentation of biomarkers is the most critical step. Usually, before the traditional laser photocoagulation, the ophthalmologists either manually mark the position of biomarkers on the color fundus image by referring to the angiography image or mark the position of biomarkers directly on the angiography image (refer to Figure 1) and then make the real-time determination of the laser photocoagulation position according to the marking position and the real fundus field of view during the operation. The time-consuming and laborious situation of manual labeling of biomarkers in the above process is an important factor to drive the related research in this paper. In addition, the exploration of automatic segmentation of biomarkers can also lay a foundation for automatic laser photocoagulation.

As far as we know, the automatic segmentation of CSCR biomarkers in the angiography image has not been widely studied. To the best of our knowledge, Ferreira has carried out relevant research so far [7] and achieved certain success. Specifically, the image processing skills such as image registration, blood vessel segmentation, optic disc detection, image inpainting, and image segmentation were combined to locate the biomarkers based on the angiography image sequence. Despite all this, some of the existing biomarker segmentation methods in fundus images and optical coherence tomography (OCT) images or target detection schemes in natural scenes can provide inspiration for our research work. The automated detection methods [8, 9] were presented to quantify the diabetic macular edema (DME) leakage area based on the angiography image sequence. With the help of image registration, image frame subtraction and vessel segmentation, the interference of normal fundus tissue such as the optic disc and vessels to segment DME biomarkers can be effectively weakened, but the difference is that the Gaussian mixture model is adopted by [9] in the final step for the biomarker segmentation, while Rabbani [8] employed the active contour segmentation algorithm to achieve the above task. Both methods performed well in the segmentation of DME biomarkers. Martinez-Costa [10] designed a scheme for leakage detection, the principle of which is to classify those pixels with a high increment in gray level within the closest area to the foveal center as the biomarkers due to retinal vein occlusion. However, this method requires manual location of the macular center. A biomarker detection framework [11] of choroidal neovascularization (CNV) was proposed, which is of great significance for the qualitative analysis of age-related macular degeneration (AMD), but more analytical techniques need to be introduced to fully count biomarker information. For studying the malarial retinopathy, a novel framework [12] was put forward to automatically detect the corresponding biomarkers, the performance of which was excellent with the introduction of saliency technique. On this basis, they developed the saliency detection method based on the intensity and compactness [13], further highlighting the advantages of the framework in the detection of biomarkers for malaria retinopathy and diabetic retinopathy.

In addition, with the development of machine learning technology, many methods based on this paradigm have emerged in the biomarker detection scenarios of various diseases, such as the discovery of biomarkers for early lung cancer diagnosis [14], predicting long-term mortality [15], the exploration of electroencephalography biomarker for Parkinson's disease classification [16], the biomarker localization of pectoralis muscle area, subcutaneous fat area and liver mass area [17], and the detection of biomarkers for multiple myeloma discrimination [18]. Besides, the machine learning method also shows its capability in the task of biomarker detection of fundus diseases. Based on the Adaboost algorithm, Tsai [19] developed an automatic biomarker segmentation method for accurate diagnosis of choroidal neovascularization. With the help of the same classifier and context knowledge, Trucco [20] achieved the localization of ischemic regions in the angiography images. To realize the joint segmentation and quantification of chorioretinal biomarkers, a residual learning-based framework integrating the atrous spatial pyramid pooling, coherent preprocessing, and postprocessing mechanisms was established, and 11 biomarkers were successfully detected [21]. Although the machine learning based method has achieved remarkable performance in all kinds of biomarker detection, this scheme is mostly driven by a large number of the labeled data, which will cause high cost of data collection and label making. Moreover, the small proportion of CSCR biomarkers in the whole image will lead to the imbalance between target pixels and background pixels, weakening the ability of machine learning method in the biomarker detection task.

However, the recently popular LRSD theory provides an effective solution for small target detection. Inspired by this theory, Gao [22] regarded the small target detection in infrared image as an optimization problem of recovering the low-rank and sparse matrices, and the scheme gained better detection performance. A LRSD-based method was put forward by Biondi [23] and able to extrapolate the sparse objects of interest in the synthetic aperture radar images. Meanwhile, the LRSD theory has also made some achievements in medical image biomarker segmentation task. Shi [24] developed an automatic segmentation method that leverages the LRSD techniques for accurate and robust detection of pathological organ from the CT images. To detect several specific types of biomarkers caused by various retinal diseases, the background of fundus image was modeled as a low-rank part, followed by the optimization algorithm and subtraction operation to achieve the final detection of biomarkers [25]. The application of the LRSD theory in the above tasks supplies us with a lot of inspiration for the task of accurate and rapid acquisition of CSCR biomarkers in this paper. The main contributions of our research are as follows: (1) we firstly introduce the multimodal fundus images into the CSCR biomarker segmentation task to avoid the interference of highlighted vessels on the location of biomarkers. (2) A simple yet effective semiautomatic localization technique is employed to reduce the search range of biomarkers, which is conducive to the improvement of segmentation efficiency. (3) To the best of our knowledge, the LRSD theory is integrated with the multimodal fundus images mentioned in (1) and the localization technique mentioned in (2) for the first time, which preliminarily realizes the construction of the baseline segmentation scheme in the CSCR biomarker segmentation task. (4) To further improve the segmentation performance of the above scheme, a joint segmentation framework consisting of the baseline scheme and RG method is designed, which is not only beneficial for the effective segmentation of CSCR biomarkers by RG, but also makes up for the defect of undersegmentation of the baseline scheme. (5) The qualitative and quantitative ablation experiments are performed to verify the effectiveness of the proposed baseline segmentation scheme and the joint segmentation framework.

The rest of the paper is organized as follows.  Section 2 presents the materials and detailed methods.  Section 3 describes experimental results and discussion.  Section 4 concludes this paper.

## 2. Materials and Methods

### 2.1. Materials

A total of 32 multimodal CSCR fundus image pairs are employed in the experiment of CSCR biomarker segmentation task, and the patient information involved in these images is processed to prevent the disclosure of privacy content. In addition, in order to utilize the multimodal information, we employ the method [26] to achieve the consistency of the spatial position between the angiography and color fundus images. Moreover, the ground truth of the CSCR biomarkers used in the experiment is annotated by three ophthalmologists to objectively test and evaluate the effectiveness of the proposed methods in the scene of CSCR biomarker segmentation.

### 2.2. Related Methods

This section focuses on the application and principle analysis of LRSD theory and RG method, which can lay the foundation for the segmentation task of CSCR biomarkers in this paper. To begin with, the LRSD-based method not only performs well in the task of target detection in natural scenes [22, 23, 27] but also has been successfully applied to the segmentation of lesions and organs in medical images [24, 25]. In general, target detection in natural scenes is realized by optimizing a paradigm in which the image background and the object to be detected are represented as *L* (i.e., low-rank matrix) and *S* (i.e., sparse matrix), respectively. In this way, target detection is cleverly transformed into the problem of solving low-rank matrix and sparse matrix in the mathematical field. The optimization problem can be expressed as follows:

(1) $$\begin{array}{c} \left(L,S\right)=\underset{L,S}{\arg\;\min\;}\mathrm{rank}\left(L\right)+\lambda {\left\|S\right\|}_{0},\text{subject to }M=L+S, \end{array}$$

where *M* is the observation matrix (i.e., the image matrix), and *λ* is the positive regularization parameter. ‖*S*‖_0_ represents the *l*_0_-norm (i.e., the number of nonzero entries in *S*). However, the solution of Eq. (1) is a NP-hard problem due to the nonconvexity of the matrix rank and the *l*_0_-norm. In this case, a relaxation scheme can replace (1), which can be written as follows:

(2) $$\begin{array}{c} \left(L,S\right)=\underset{L,S}{\arg\;\min\;}{\left\|L\right\|}_{*}+\lambda {\left\|S\right\|}_{1},\text{subject to }M=L+S, \end{array}$$

where ‖*L*‖_∗_denotes the nuclear norm of the matrix *L* (i.e., the sum of singular values of *L*), and ‖*S*‖_1_ is the *l*_1_-norm that represents the sum of the absolute values of *S* (i.e., ‖*S*‖_1_ = ∑_*i*,*j*_ | *S*_*ij*_∣).Under certain assumptions, *L* and *S* can be obtained based on the robust principal component analysis (RPCA) [28].

Furthermore, as a simple yet effective image segmentation technique, the RG method has been extensively utilized in the field of medical image processing and analysis, such as the segmentation of chondroblastoma in the X-ray image [29] and the brain segmentation in the CT and MRI images [30, 31]. The basic principles of the algorithms in the above applications are consistent, which divide the image into different regions according to the similarity between pixels, and the specific implementation steps involve the selection of seed points, the construction of growth rules, and the design of termination conditions. Although the RG method has a good performance in the medical image segmentation task, its accurate and fast seed point selection operation is still an issue to be overcome, which is of great significance to improve its segmentation quality and efficiency. In view of this situation, we propose a joint segmentation framework to make the RG method perform more remarkable in the segmentation task of CSCR biomarkers.

### 2.3. The Proposed Methodology

**Figure 2:**
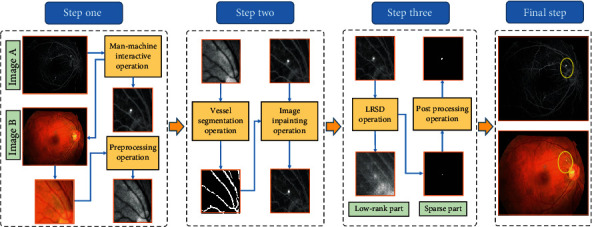
The schematic diagram of the baseline segmentation scheme.

The successful application of the LRSD theory and RG method provides inspiration for us to carry out the CSCR biomarker segmentation task. On this basis, we further extend the above methods from the perspective of technique integration and apply the proposed methods to the segmentation of CSCR biomarkers. In general, a baseline segmentation scheme integrating multimodal fundus images, semiautomatic localization technique, and LRSD theory is presented, which initially achieves the goal of rapid acquisition of biomarker location information. The specific steps are shown in Figure 2.

Step one: this step consists of the man-machine interactive and preprocessing operations. In order to speed up the location of biomarkers, the man-machine interactive operation (i.e., semiautomatic localization technique) is implemented on the angiography images, which is to initially lock the biomarker area through manual box selection mode and can thus avoid the mathematical operation on the whole image matrix. The location information of the box will be transferred to the color fundus image synchronously to ensure that the same area can be extracted. After that, the preprocessing operation composed of the green channel separation and contrast limited adaptive histogram equalization (CLAHE) is applied to the color fundus image block to obtain the preprocessed image block *I*_*c*_ for facilitating the subsequent vessel segmentation.

Step two: in this procedure, the vessel segmentation operation and the image inpainting operation are performed continuously. Firstly, the Laplacian of Gaussian (LoG) operation is performed on the preprocessed image block *I*_*c*_ in the previous step to obtain the blood vessels. The specific formulas can be expressed as follows:

(3) $$\begin{array}{c} \text{LoG}\left(r,c\right)=-\frac{1}{{\pi \sigma }^{4}}\left[1-\frac{r^{2}+c^{2}}{2\sigma^{2}}\right]e^{-\frac{r^{2}+c^{2}}{2\sigma^{2}}}, \end{array}$$

(4) $$\begin{array}{c} G\left(x,y\right)=\overset{k}{\underset{r=-k}{\sum }}\overset{l}{\underset{c=-l}{\sum }}\text{L}\text{oG}\left(r,c\right)Ic\left(x+r,y+c\right), \end{array}$$

where *σ* denotes the standard deviation with a value of 3, and (*r*, *c*) is the coordinate of the element in the LoG filter. *k* and *l* are the nonnegative integers that are both set to 3 in this paper. *G*(*x*, *y*) represents the spatial filtering result of image *I*_*c*__,_ and (*x*, *y*) is the coordinate of the corresponding element. Then, the final vessel mask can be acquired though a simple postprocessing mainly consisting of the binarization and small area removal processes. On this basis, the image inpainting technique [32] is adopted to weaken the highlighted vessel area in the angiography image block *I*_*a*_ and fill it with the surrounding background pixels.

Step three: after the image inpainting operation, the CSCR biomarker segmentation model is then established based on the LRSD theory and the inpainting image *G*_*P*_. Considering that the fundus image is not absolutely pure, the noise in the image needs to be taken into account in the modeling process. Thus, the image *G*_*P*_ can be represented as Eq. (5).

(5) $$\begin{array}{c} Gp=L+S+N, \end{array}$$

where *N* is the noise part of the image *G*_*P*_ (i.e., the observation matrix *M*) and is assumed to be independent and identically distributed(i.e., i.i.d.) in this paper. According to the conditions in [33], *L* and *S* can be achieved with the assumption ‖*N*‖_*F*_ ≤ *δ* for some *δ* > 0, and then Eq. (2) can be converted into the following relaxed version:

(6) $$\begin{array}{c} \left(L,S\right)=\underset{L,S}{\arg\;\min\;}{\left\|L\right\|}_{*}+\lambda {\left\|S\right\|}_{1},\text{subject to }{\left\|Gp-L-S\right\|}_{F}\leq \delta . \end{array}$$

The dual form of Eq. (6) can be expressed as follows:

(7) $$\begin{array}{c} \left(L,S,N\right)=\underset{L,S,N}{\arg\;\min\;}{\left\|L\right\|}_{*}+\lambda {\left\|S\right\|}_{1}+\frac{1}{2\mu }{\left\|N\right\|}_{F}^{2},\text{subject to }Gp=L+S+N, \end{array}$$

where ‖*N*‖_*F*_ denotes the Frobenius norm (i.e., ${\left\|N\right\|}_{F}=\sqrt{\sum_{i,j}N_{ij}^{2}}$). *μ* is a positive weight parameter. As usual, the Accelerated Proximal Gradient (APG) [34] is applied to recover *L* and *S* in this paper. Besides, in order to solve Eq. (7) more effectively, the patch-image model [22] is employ to reconstruct *G*_*P*_, and the corresponding patch size and sliding step are set to 50 × 50 and 10, respectively. In view of the stability and accuracy of the APG method in the infrared target detection task, the experimental setup of this part is consistent with [22] in which the detailed parameters can be found. Then, the postprocessing operations mainly including the searching and sorting of connected regions are performed on *S* to promote the generation of the final CSCR biomarker block *S*_*P*_.

Final step: although the semiautomatic localization technique accelerates the segmentation efficiency of CSCR biomarkers, it will cause the spatial positions of biomarkers obtained in the previous step to be inconsistent with their original positions. Therefore, in this step, the initial box position information is utilized to restore *S*_*P*_ to the original color fundus and angiography images, which is essential to provide effective position information of biomarkers for the ophthalmologists and automatic laser equipment.

**Figure 3:**
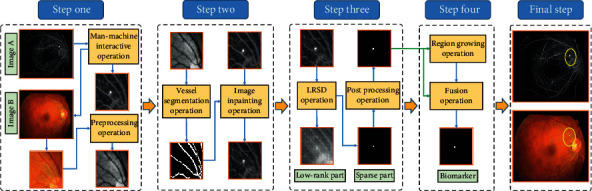
The schematic diagram of the joint segmentation framework.

In the above scheme, the introduction of multimodal fundus images weakens the interference of the highlighted vessels in the angiography image to the location of biomarkers, and the utilization of semiautomatic localization technique improves the efficiency of biomarker localization. Meanwhile, the LRSD theory transforms the task of biomarker segmentation into the problem of low-rank and sparse matrix decomposition, which is not only conducive to the effective location locking of biomarkers but also accelerates the efficiency of biomarker segmentation to a certain extent. Nevertheless, this scheme may cause the problem of undersegmentation, which leads to the inaccurate segmentation results, and thus stimulates the further design of a joint segmentation framework (refer to Figure 3 for the framework). Specifically, we combine the RG method with the above scheme. On the one hand, the LRSD theory is employed to provide the initial seed points for the RG method to ensure that it can obtain effective biomarkers. On the other hand, the segmentation results obtained by RG and LRSD are fused to make up for the undersegmentation defect of the baseline segmentation scheme.

As shown in Figure 3, after the step three of baseline segmentation scheme, the step four is followed closely. Firstly, the position information of biomarkers in *S*_*P*_ is extracted to provide the initial region growing point for the RG method. Secondly, the biomarkers are automatically segmented based on the RG method under the condition of a given threshold. Then, the segmentation results of the two schemes are fused to get the final biomarkers. The process can be formulated as follows:

(8) $$\begin{array}{c} \left(S_{d}^{x},S_{d}^{y}\right)=\underset{S_{d}^{x},S_{d}^{y}}{\arg\;\max}Spd\left(1:K1,1:K2\right),d\in \left[1,K\right], \end{array}$$

(9) $$\begin{array}{c} Rpd=\text{RG}\left(Ia,S_{d}^{x},S_{d}^{y},T\right), \end{array}$$

(10) $$\begin{array}{c} FS=\overset{K}{\underset{d=1}{\sum }}\left(Spd+Rpd\right), \end{array}$$

where *K* is the number of the biomarkers in the final CSCR biomarker block *S*_*P*_, and *S*_*Pd*_ corresponds to the image block only containing the *d*-th biomarker. The size of *S*_*P*_ is *K*_1_ × *K*_2_, and (*S*_*d*_^*x*^, *S*_*d*_^*y*^) denotes the position information of the *d-*th biomarker in *S*_*P*_. *T* is the threshold that is varied from 0.1 to 0.2 with a step size of 0.02. *R*_*pd*_ represents the segmented result of the *d*-th biomarker by the RG method. *F*_*S*_ is the fusion result of the biomarkers acquired by the baseline segmentation scheme and RG method. Then, the operation in the final step of the baseline segmentation scheme is also performed on *F*_*S*_ to obtain the final CSCR biomarkers. The pseudocode of step four is as follows:

## 3. Results and Discussion

### 3.1. The Evaluating Indicators and Experimental Settings

In order to quantitatively evaluate the performance of baseline scheme and joint scheme in the CSCR biomarker segmentation task, sensitivity, F1-score, accuracy, and specificity are introduced as the evaluation indicators. The details are as follows:

(11) $$\begin{array}{c} \text{Sensitivity}=\text{TP}/\left(\text{TP}+\text{FN}\right), \end{array}$$

(12) $$\begin{array}{c} \text{F}1‐\text{score}=2\text{TP}/\left(2\text{TP}+\text{FN}+\text{FP}\right), \end{array}$$

(13) $$\begin{array}{c} \text{Accuracy}=\left(\text{TP}+\text{TN}\right)/\left(\text{TP}+\text{FN}+\text{TN}+\text{FP}\right), \end{array}$$

(14) $$\begin{array}{c} \text{Specificity}=\text{TN}/\left(\text{TN}+\text{FP}\right), \end{array}$$

where the manually annotated biomarker pixels that are correctly segmented are defined as true positives (TP) and those that are wrongly segmented are false negatives(FN). Similarly, the manually annotated nonbiomarker pixels that are correctly identified are true negatives (TN), and the wrongly specified nonbiomarker pixels are false positives (FP). In addition, the ablation experiments are carried out carefully to show the rationality and effectiveness of the proposed schemes.

For convenience, the baseline segmentation scheme in this paper is represented by LRM, and the version of this scheme without the multimodal technique is marked as LR. Meanwhile, the proposed joint segmentation framework is denoted by LRM + R, and its version without the fusion module is represented by LRM⟶R, which means that the LRSD is only applied to offer the initial seed points for the RG method.

### 3.2. The Discussion of Segmentation Results of Baseline Scheme

**Figure 4:**
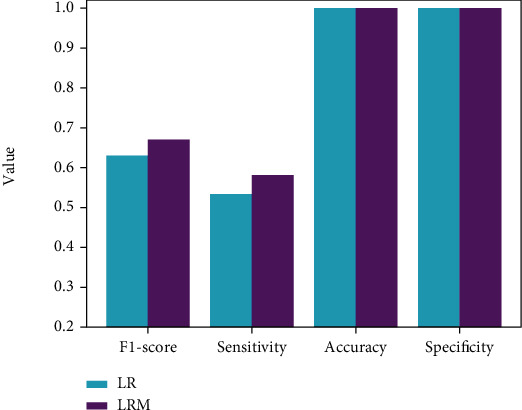
The comparison of LRM and LR.

This section analyzes the performance of baseline segmentation scheme LRM. As shown in Figure 4, when the multimodal technique is not adopted, LR is significantly inferior to LRM in terms of F1-score and sensitivity indicators, which also implies the effectiveness and importance of introducing the multimodal fundus images into the CSCR biomarker segmentation task. Additionally, it can be clearly found that both LR and LRM have achieved more than 90% in the other two indicators, and the difference of the same indicators is not obvious. This is mainly credited to the introduction of the semiautomatic location technique, which reduces the detection range of CSCR biomarkers and thus greatly decreases the false positives.

### 3.3. The Discussion of Segmentation Results of Joint Framework

**Figure 5:**
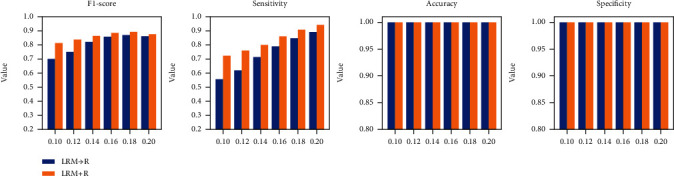
The comparison of LRM⟶R and LRM + R under the condition of various thresholds.

Figure 5 shows the performance of LRM⟶R and LRM + R in the CSCR biomarker segmentation scene. It should be noted that this figure is the overall performance of the two schemes on 29 image pairs. The reason is that the two schemes cannot work well on the other three image pairs when the threshold is set to 0.2, which also contributes to the threshold selection of the experiment from one of the perspectives. Further, with the threshold increasing from 0.1 to 0.18, the two schemes reveal an increasing trend in terms of F1-score and sensitivity indicators and obtain the best segmentation results at 0.18. Meanwhile, LRM + R is better than LRM⟶R in these two indicators, which conveys the necessity of the fusion technique. For the other two indicators, the two schemes perform well, the reason of which is consistent with LR and LRM.

**Figure 6:**
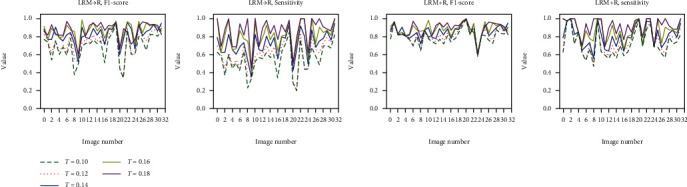
The comparison of LRM⟶R and LRM + R on each image with various thresholds.

According to the above analysis, 0.2 is not an appropriate threshold for the CSCR biomarker segmentation task. In view of this, we observe the specific performance of the two schemes on 32 image pairs based on the other five thresholds. On the whole, it can be seen from Figure 6 that the performance of the two schemes is improved with the increase of threshold in terms of F1-score and sensitivity indicators. However, with the participation of the fusion technique, LRM + R can significantly create a smaller gap under different threshold conditions compared with LRM⟶R, which reveals that the joint segmentation framework can lower the impact of threshold parameters on the segmentation performance of the model and thus alleviates the pressure of threshold selection to a certain extent. Moreover, the fluctuations of the two indicator values of each image pair based on the joint segmentation framework are less than those based on LRM⟶R in most cases and show that LRM + R possesses better robustness in the biomarker segmentation task.

### 3.4. The Display and Discussion of Ablation Experiment Results

**Figure 7:**
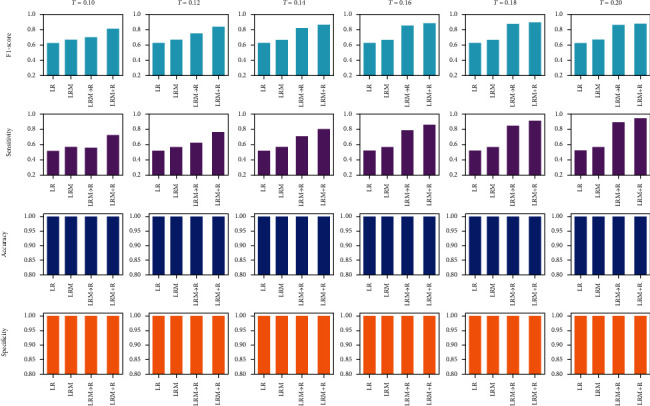
The overall comparison of the four schemes.

**Figure 8:**
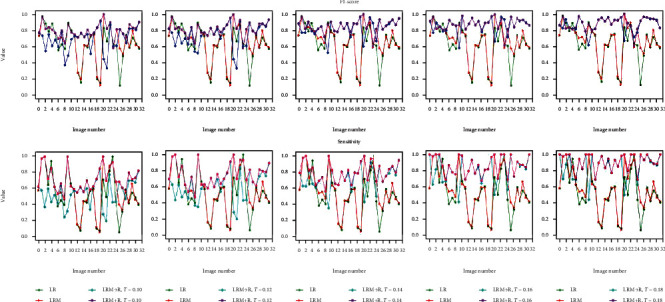
The performance of four schemes on each image.

This section shows and discusses the overall performance of the four schemes.  Figure 7 is the statistical results of four indicators of ablation experiment on 29 images, in which the performance of four schemes can be clearly displayed. On the one hand, the introduction of semiautomatic localization technique promotes the four schemes to obtain higher accuracy and F1-score values. On the other hand, the segmentation ability of the joint segmentation framework designed on the basis of LRM is further enhanced under different thresholds, and the two indicator values of this framework exceed 80% when the threshold is greater than 0.12. Simultaneously, the independent performance (refer to Figure 8) of the four schemes on 32 images not only further demonstrates the effectiveness of the baseline segmentation scheme but also proves the necessity of further upgrading it to obtain LRM + R.

**Figure 9:**
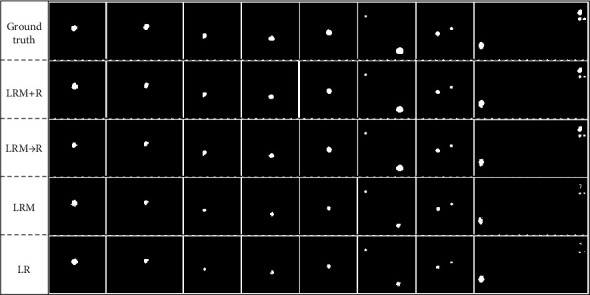
The segmentation results of CSCR biomarkers.

Furthermore, in order to convey the ablation experiment results more intuitively, some of the CSCR biomarker segmentation results achieved by the four schemes with the threshold of 0.18 are shown in Figure 9. It can be found that LRM is better than LR in most cases, but it has the defect of undersegmentation when compared with the ground truth. Although the LRM⟶R scheme avoids the tedious matter of manually selecting seed points and almost exceeds the LRM in the actual segmentation task, the threshold factor makes it not always perform well. In this case, the proposed joint segmentation framework improves LRM⟶R partly, in which the fusion technique based on the LRSD theory and RG method increases the true positives.

Finally, we discuss the CSCR biomarker segmentation results in the ablation experiments quantitatively.  Table 1 records the average indicator values of 32 images segmentation results acquired by the four schemes under five thresholds (i.e., 0.1, 0.12, 0.14, 0.16 and 0.18). Of note, since the previous qualitative analysis has shown that the other two indicators of the four schemes are very high and lack of significant distinction, only the Accuracy and F1-score are taken into consideration here. As shown in Pseudocode 1, the proposed baseline segmentation scheme exceeds LR by 4.026% and 4.7329%, respectively, in the two indicators, which is an affirmation of the multi-modal technique in weakening the interference of highlighted vessels to the location of CSCR biomarkers in the angiography images. On this basis, LRM → R works well under different thresholds and achieves more than 86% of the indicator value when the threshold is set to 0.18, which means that LRSD can provide an effective initial seed points for the RG method and then promote the segmentation of real CSCR biomarkers. However, this scheme may be limited by the thresholds, thus resulting in insufficient segmentation of the CSCR biomarkers. Fortunately, experiments show that the joint segmentation framework can alleviate this problem. It can be found that LRM + R has made significant progress, and the indicator values at the maximum threshold are 88.3972% and 91.5447%, respectively, which are 1.7374% and 5.5111% higher than those of LRM⟶R and 21.3741% and 33.4602% higher than those of LRM. This demonstrates the superiority of the proposed joint segmentation framework.

## 4. Conclusions

In this paper, two CSCR biomarker segmentation methods are proposed to locate the leakage area efficiently and accurately, which can assist laser surgery in the treatment of chronic CSCR. Firstly, a baseline segmentation scheme integrating the multimodal fundus images, semiautomatic localization technique, and LRSD theory is proposed, which is not only the first attempt of LRSD in locating the CSCR leakage area, but also the preliminary realization of rapid acquisition of these biomarkers. Then, a joint segmentation framework is further designed to improve the above method, aiming at enabling the LRSD to supply the effective seed points to the RG method and making up for the defect of undersegmentation of baseline scheme. Qualitative and quantitative experiments demonstrate the feasibility of the baseline scheme to obtain the biomarkers and the effectiveness of the joint segmentation framework in improving the segmentation quality. In the future, the fully automatic CSCR biomarker segmentation method equipped with high segmentation quality and efficiency will be further explored based on the above research to fulfill the requirements of real-time locking CSCR biomarkers in automatic laser surgery.

## Figures and Tables

**Pseudocode 1:**
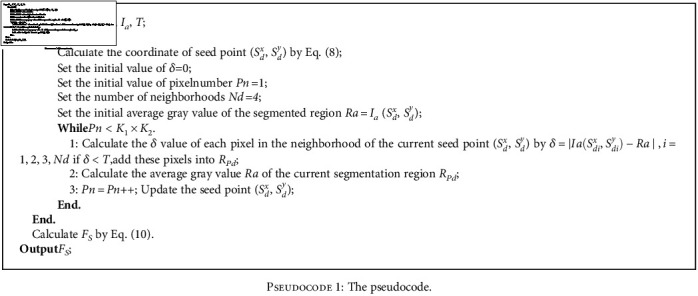
The pseudocode.

**Table 1:** The average values of F1-score and accuracy indicators.

Methods	T	F1-score/%	Sensitivity/%
LRM + R	0.10	80.2341	72.2436
	0.12	82.4836	75.5831
	0.14	85.7589	80.5158
	0.16	88.3595	86.9361
	0.18	88.3972	91.5447
LRM⟶R	0.10	69.3491	54.8737
	0.12	74.0886	60.8251
	0.14	82.6932	71.8034
	0.16	86.6628	80.4348
	0.18	86.6598	86.0336
LRM		67.0231	58.0845
LR		62.9971	53.3516

## Data Availability

The data used and analyzed in our research are available from the corresponding author upon request.
